# Adult-onset Minimal Change Disease with IgA Nephropathy and Hepatitis C

**DOI:** 10.7759/cureus.2207

**Published:** 2018-02-19

**Authors:** Waliul Chowdhury, Tahira Sabeen Saleem, Muhammad Uzair Lodhi, Intekhab Askari Syed, Hafiz Imran Iqbal, Mustafa Rahim

**Affiliations:** 1 Medical Student, Department of Medicine, Raleigh General Hospital, Beckley, Wv; 2 Department of Medicine, Raleigh General Hospital, Beckley, Wv; 3 Nephrologist, Department of Medicine, Raleigh General Hospital, Beckley, Wv; 4 Assistant Clinical Professor of Internal Medicine, West Virginia University School of Medicine

**Keywords:** minimal change disease, iga nephropathy, hepatitis c

## Abstract

Minimal change disease (MCD) is one of the most common causes of nephrotic syndrome in children, leading to heavy proteinuria and edema. However, it is not as common in adults. Adult-onset minimal change disease with IgA nephropathy is rare. The initial presentation of heavy proteinuria and edema with effacement of podocytes on electron microscopy (EM) should lead the physician to suspect minimal change disease regardless of age. We present a 44-year-old male patient with a history of hepatitis C virus (HCV) who presented with sudden onset of lower extremity edema and 6.6 grams (g) of proteinuria per day.

## Introduction

Minimal change disease (MCD) is one of the most common causes of nephrotic syndrome in children, but less common in adults [1]. The cause of nephrotic syndrome is mostly idiopathic, but it can also be triggered by an infection or immune stimulus [2]. Dysregulation of the immune system along with modifications of the podocytes is thought to play a role in any type of nephrotic syndrome. This changes the integrity of the glomerular basement membrane (GBM) filtration barrier, causing proteinuria [1-2]. The biopsy findings of MCD include no visible changes on light microscopy (LM) and effacement of the foot processes on electron microscopy (EM) [1]. IgA nephropathy will show positive antibody staining for IgA on immunofluorescence [3]. Physicians should rule out the possibility of MCD in adults even though it is rare. When physicians suspect nephrotic syndrome in patients they should obtain a renal biopsy to make their diagnosis more specific. They should also obtain a detailed history to analyze whether secondary conditions are triggering the event. We present a 44-year-old male patient with a past medical history of hepatitis C infection presenting with a sudden onset of lower extremity edema and heavy proteinuria.

## Case presentation

History and physical examination

A 44-year-old male presented to the hospital with swelling in the lower extremities lasting three months. It was initially around the ankle, but it got worse and extended to the thighs over the last few weeks. The patient did not have a primary care provider (PCP) and the last time he saw a doctor was several years ago. He denied any history of kidney disease, diabetes, hypertension or congestive heart failure. He was currently not taking any medication. He had no known drug allergies. His past surgical history was significant for appendectomy in early childhood. He denied smoking and his last drink of alcohol was four months ago. He admitted to using intravenous (IV) drugs in the past, and his last IV drug use was two months ago. He denied any shortness of breath or chest pain.

On physical examination, the patient was not in acute distress. His blood pressure was 133/72, heart rate was 86 beats per minute (bpm), oxygen saturation was 97%. His head was normocephalic and atraumatic. The pupils were equal and reactive. There was no thyromegaly, lymphadenopathy, or jugular venous distention. There was a systolic murmur heard upon heart examination. His abdomen was distended but soft. There was significant dependent edema in his lower extremities which extended to the genital area.

Hospital course

His urinalysis showed significant proteinuria of +3 on urine dipstick and his 24-hour total urinary protein level was significantly high of 12,136 mg/dl, as shown in Table 1. The workup for proteinuria was negative, which included antinuclear antibody (ANA), antineutrophil cytoplasmic antibody (ANCA) panel, antidouble stranded DNA, complement 3 (C3) level, complement 4 (C4) level, and free serum kappa to lambda ratio with serum protein electrophoresis. His complete metabolic panel was significant for a low albumin level of 1.0 g/dl, a low total protein (PEP) of 4.5 g/dl, and a low 25-hydroxy vitamin D level of 6.3 g/dl. The metabolic panel is shown below in Table 2. His complete blood count showed anemia with a hemoglobin of 8.7 gm/dl, as shown in Table 3. His renal ultrasound findings were unremarkable with no signs of hydronephrosis or renal calculi. There was no mass or cyst. Vascular flow was also normal.

**Table 1:** Urinalysis result.

Test	Result
Urine appearance	Slightly hazy
Urine protein	3+
Urine blood	2+
Urine epithelial cells	2-5/high power field
Urine bacteria	1+
Hyaline casts	1-3/high power field
Urine total protein 24 hour	12,136 mg/24 hours

**Table 2:** Metabolic panel. LDL: Low-density lipoprotein; VLDL: Very low-density lipoprotein.

Test	Result
Sodium	139 mEq/L
Potassium	4.1 mEq/L
Calcium	7.5 mg/dL
Total protein (PEP)	4.5 g/dL
Alpha-2-globulins	1.3 g/dL
Triglycerides	223 mg/dL
LDL cholesterol	204 mg/dL
VLDL cholesterol	45 mg/dL
Vitamin D 25-hydroxylase	6.3 ng/dL
Vitamin D3	6.2 ng/dL

**Table 3:** Complete blood count.

Test	Result
White blood cells	14.2 x 10^9^/L
Red blood cells	3.09 x 10^12^/L
Hemoglobin	8.7 gm/dL
Mean corpuscular volume	84 gm/dL
Platelet count	335 x 10^9^/L
Vacuolated neutrophils	Few
Anisocytosis	1+

**Figure 1:**
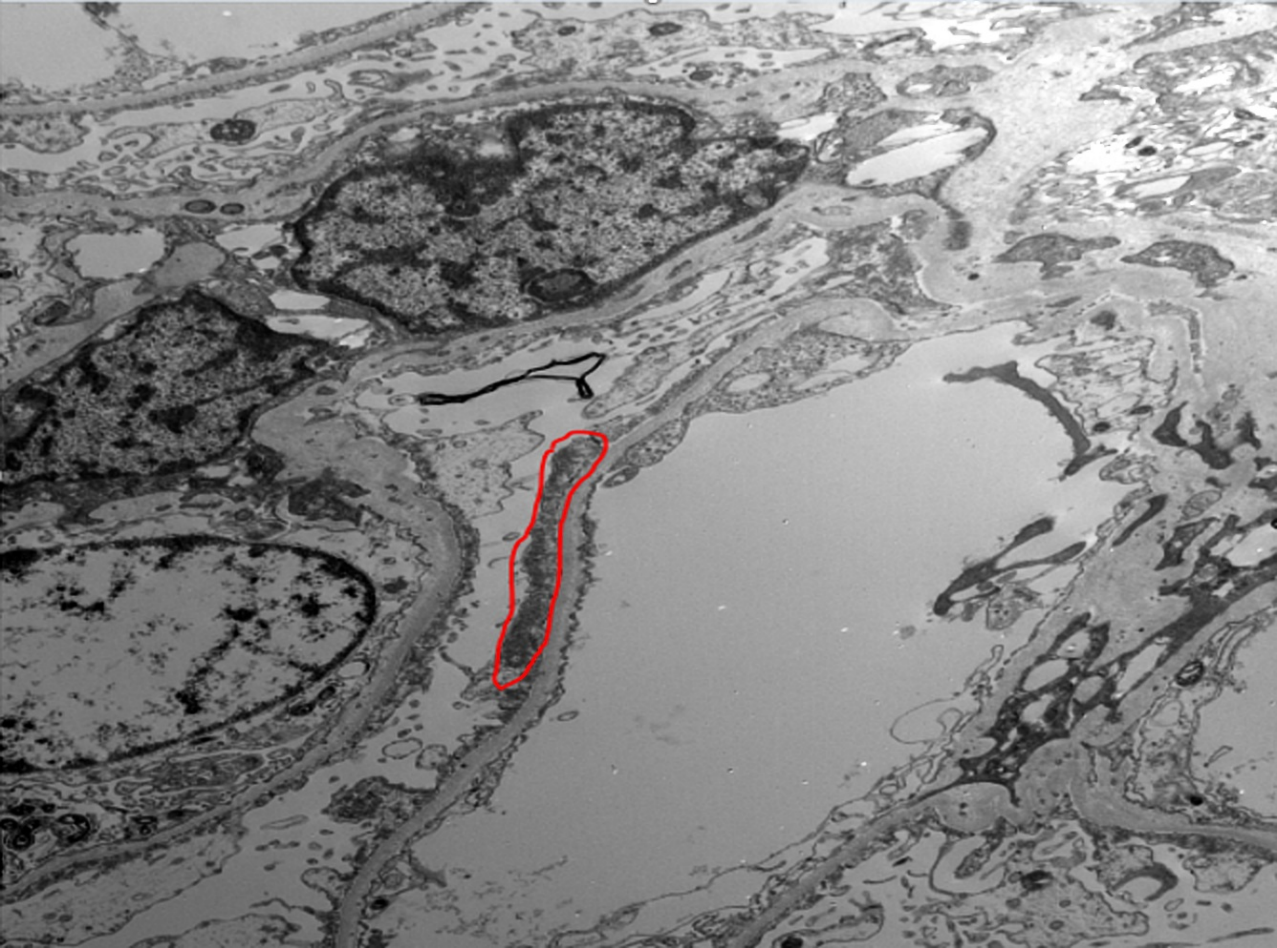
Electron microscopy of the patient showing effacement of the podocytes. Red circle = effacement of the podocytes

A renal biopsy was also ordered. On electron microscopy, four survey sections were examined. There were six glomeruli present. None of the glomeruli were globally sclerosed. Ultrastructural examination showed extensive and diffuse effacement of the foot processes of the visceral epithelial cells, shown in Figure 1. Electron-dense deposits were not present along the capillary walls or tubular basement membrane. The glomerular basement membrane appeared to be of normal thickness. Basement membrane splitting and lamellations were not present. The endothelial cells did not contain tubuloreticular inclusions.

**Figure 2:**
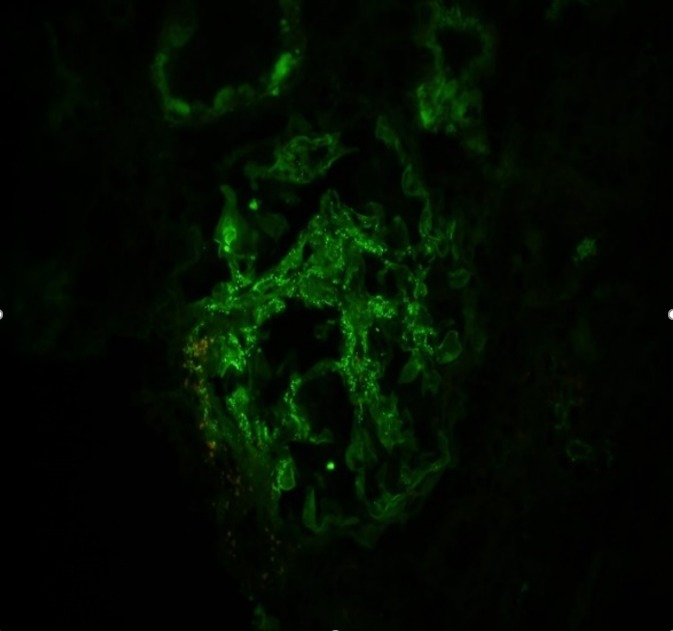
Immunofluorescence (IF) of the patient. This IF shows mild mesangial staining for IgA (2+), C3 (trace), kappa light chains (+1), and lambda light chains (+1). The depositions are linear.

On immunofluorescence, there were up to eight glomeruli present. None of the glomeruli were sclerosed. The glomeruli showed mild mesangial staining for IgA (+2), trace amounts of C3, kappa light chains (+1), and lambda light chains (+1), as shown in Figure 2. The glomeruli were negative for IgG, IgM, albumin, and fibrinogen.

On light microscopy, there were approximately 21 glomeruli present. There was only a mild increase in mesangial matrix and minimal segmental increase in cellularity, mostly due to mononuclear cells. The glomeruli showed no evidence of crescents, fibrinoid necrosis, thrombosis, or endocapillary hypercellularity. Basement membrane spikes, pinholes, or double contours were not present along the capillary walls. Some of the tubules showed degenerative changes with distention and flattening of the epithelium. There was minimal focal interstitial inflammation present. There was also a mild tubular atrophy (10-20%) and interstitial fibrosis present. The arteries showed mild sclerosis of the intima. However, there were no signs of thrombosis, emboli or arteritis.

After optimizing the volume status with intravenous diuretics and confirmation of biopsy findings he was started on prednisone 1 mg/kg along with prophylactic proton pump inhibitors. Diuretics were changed from oral furosemide 40 mg twice a day to metolazone 2.5 mg once a day upon discharge from the hospital.

Differential Diagnosis

This patient had heavy proteinuria with edema and hypoalbuminemia, which lead us to the diagnosis of nephrotic syndrome [1-3]. However, physicians should rule out the other types of nephrotic syndrome from the biopsy results before making a final diagnosis. The combined diagnosis of MCD with mild IgA nephropathy in this patient was supported by diffuse podocyte effacement on electron microscopy and distribution of IgA deposits on immunofluorescence, respectively [1-3].

Effacement of foot processes can also be seen on electron microscopy in focal segmental glomerular sclerosis (FSGS) [4]. There continues to be a debate as to whether MCD and FSGS fall within a spectrum of the same disease or whether they represent separate pathogenetic entities. Other than descriptive changes on light microscopy, the two entities can be separated based on clinical and pathological features. MCD is characterized by complete foot process effacement on electron microscopy and an excellent response to steroids. In contrast, patients with FSGS usually respond poorly to steroid treatment [4].

## Discussion

The pathophysiology of nephrotic syndrome is still not fully understood. Cytokines can mediate damage to the podocytes [1]. This leads to decreased synthesis of polyanions along the glomerular basement membrane [1-2]. Polyanions play a role in forming the normal negative charge barrier which repels the filtration of macromolecules, mainly the albumin. This decreased charge barrier is what leads to proteins leaking across the glomerular filtration barrier, causing heavy proteinuria [2].

Having patients with IgA nephropathy and minimal change disease together is rare. However, studies have shown IgA nephropathy and minimal change disease to occur together in a small amount of patients [3]. A retrospective study by Herlitz, et al. reviewed cases of IgA nephropathy diagnosed from 2004 to 2011 that had evidence of nephrotic range proteinuria and mild IgA nephropathy. They identified cases that lacked endocapillary proliferation or segmental sclerosis. The study consisted cases from 17 patients, 15 of whom were adults. The median serum creatinine was 0.9 mg/dl, 24-hour urinary protein level was 8.0 g/dl. Fourteen patients were completely nephrotic, and three patients had two of the three criteria required to diagnose nephrotic syndrome. Electron microscopy showed mesangial deposits and significant foot process effacement. These patients were given corticosteroids for treatment. Over a 20-month follow-up, 14 patients completely responded to treatment and three patients had a partial response. There was a relapse of nephrotic syndrome in nine out of 17 patients [3]. This study showed that rare cases of IgA nephropathy with minimal change disease can be diagnosed in adults by their clinical presentation, biopsy findings, treatment response, and outcome.

Urinary excretion of cluster of differentiation 80 (CD80) can be used to differentiate patients with MCD from FSGS. Studies have shown urinary CD80 to be significantly increased in MCD patients compared to patients with FSGS [4]. For example, Garin, et al. conducted a study on 17 patients with MCD and 22 patients with FSGS and measured their urinary CD80 excretion. The urinary CD80 excretion was measured using an enzyme-linked immunosorbent assay (ELISA) and its size was determined using a western blot. They found a significant increase in urinary CD80 excretion in patients with MCD compared to patients in remission or with FSGS [4]. This study shows that checking for increases in urinary CD80 can be a useful tool to distinguish between MCD and FSGS.

The identity of circulating permeability factor remains unknown, however, hemopexin has been under study and is a good candidate [5]. Savin, et al. did a study to show that hemopexin is a protease that seems to play a role in altering glomerular endothelial and podocyte function. In glomerular endothelial cells, it reduces the endothelial glycocalyx layer and increases diffusion of albumin. When hemopexin was injected in rats it caused proteinuria and effacement of podocytes [5]. Knowing the identity of circulating permeability could play a significant role in targeted therapies for nephrotic syndrome in the future.

Checking for increases in IgE levels may have a significant predicting power in detecting minimal change disease [6]. Shao, et al. composed a retrospective study of 76 adult nephrotic syndrome patients with heavy proteinuria (> or = 3.5 g/day) and normal creatinine levels. The renal biopsy results were analyzed from January 2006 to January 2007. Twenty-four predictive variables were retrospectively gathered by reviewing the charts a day before the renal biopsy. These included IgG, IgA, IgM, and IgE, as well as demographic variables. The prevalence of MCD was found in 21 out of 76 patients in this group. An independent student t-test was done on this patient group with MCD which showed three out of 24 variables being statistically significant (p < 0.05). Serum IgE levels showed excellent discriminative power in predicting MCD (area under the curve = 0.868 +/- 0.053; p = 0.001) [6]. This study showed that checking for elevations in serum IgE levels could be a straightforward and cost-effective screening tool in predicting MCD. However, a renal biopsy should still be done to confirm the diagnosis.

A possible correlation between HCV and IgA nephropathy could be the Fc fragment of IgA receptor (FCAR), which is a human gene coding for cluster of differentiation 89 (CD89). CD89 can trigger IgA-mediated immune responses and may play a role in IgA clearance [7]. Watanabe, et al. investigated if promoter polymorphisms of the FCAR gene could be associated with hepatitis C infection. Two single nucleotide polymorphisms (SNPs) were studied and sequenced in 177 Japanese patients with chronic HCV infection. They showed that the two genotypes were shown significantly more in these patients compared to 210 healthy control patients. This shows that the FCAR gene coding for CD89 could be associated with chronic hepatitis C infection and progression [7].

Another study by Moresco, et al. investigated the use of urinary CD89 as a biomarker for IgA nephropathy and Henoch-Schönlein purpura nephritis (HSPN). They did a multicenter study in which 169 patients with active IgA nephropathy and HSPN were enrolled. They found that urinary CD89 was lower in patients with both active nephropathies compared to patients in complete remission [8]. Both studies showed that CD89 can be used as a marker on a wide scale in the future [7-8].

## Conclusions

Adult-onset minimal change disease with superimposed mild IgA nephropathy and a history of hepatitis C infection is a rare combination. It is important for physicians to consider a renal biopsy in patients with hepatitis C and renal disease because it could correlate to glomerulopathies with or without cryoglobulinemia. Even though IgA nephropathy is more commonly associated with hepatitis B, it can also be seen with hepatitis C. Even though MCD in adults is rare, it should still be considered as a differential diagnosis by physicians. Although we discussed possible markers of IgA nephropathy and minimal change disease, a biopsy is still the gold standard to confirm the diagnosis.
